# Sterilization of liquid foods by pulsed electric fields–an innovative ultra-high temperature process

**DOI:** 10.3389/fmicb.2015.00400

**Published:** 2015-05-06

**Authors:** Kai Reineke, Felix Schottroff, Nicolas Meneses, Dietrich Knorr

**Affiliations:** ^1^Quality and Safety of Food and Feed, Leibniz Institute for Agricultural EngineeringPotsdam, Germany; ^2^Department of Food Biotechnology and Food Process Engineering, Technische Universitaet BerlinBerlin, Germany; ^3^Corporate Technology, Buehler AGUzwil, Switzerland

**Keywords:** pulsed electric fields, sterilization, endospore-forming bacteria, milk, inactivation mechanisms

## Abstract

The intention of this study was to investigate the inactivation of endospores by a combined thermal and pulsed electric field (PEF) treatment. Therefore, self-cultivated spores of *Bacillus subtilis* and commercial *Geobacillus stearothermophilus* spores with certified heat resistance were utilized. Spores of both strains were suspended in saline water (5.3 mS cm^−1^), skim milk (0.3% fat; 5.3 mS cm^−1^) and fresh prepared carrot juice (7.73 mS cm^−1^). The combination of moderate preheating (70–90°C) and an insulated PEF-chamber, combined with a holding tube (65 cm) and a heat exchanger for cooling, enabled a rapid heat up to 105–140°C (measured above the PEF chamber) within 92.2–368.9 μs. To compare the PEF process with a pure thermal inactivation, each spore suspension was heat treated in thin glass capillaries and *D*-values from 90 to 130°C and its corresponding *z*-values were calculated. For a comparison of the inactivation data, *F*-values for the temperature fields of both processes were calculated by using computational fluid dynamics (CFD). A preheating of saline water to 70°C with a flow rate of 5 l h^−1^, a frequency of 150 Hz and an energy input of 226.5 kJ kg^−1^, resulted in a measured outlet temperature of 117°C and a 4.67 log_10_ inactivation of *B. subtilis*. The thermal process with identical *F*-value caused only a 3.71 log_10_ inactivation. This synergism of moderate preheating and PEF was even more pronounced for *G. stearothermophilus* spores in saline water. A preheating to 95°C and an energy input of 144 kJ kg^−1^ resulted in an outlet temperature of 126°C and a 3.28 log_10_ inactivation, whereas nearly no inactivation (0.2 log_10_) was achieved during the thermal treatment. Hence, the PEF technology was evaluated as an alternative ultra-high temperature process. However, for an industrial scale application of this process for sterilization, optimization of the treatment chamber design is needed to reduce the occurring inhomogeneous temperature fields.

## Introduction

Based on the consumers' demand for fresh, durable and safe foods it is obligatory for the food industry to preserve their products at the best. To obtain products with longest possible shelf-lives, inactivation of spores is inevitable. This is usually achieved by thermal sterilization, a process mostly realized through autoclaving at 121°C for approximately 15 min. This eliminates all microorganisms and spores although valuable food ingredients are destroyed (Jaeger et al., 2014a). Therefore, a innovative preservation method is the application of pulsed electric fields (PEF), which might by suitable for sterilization of liquid food (Siemer et al., 2014a,b).

Currently, PEF processing is applied in the food industry for enhancing mass transport, increasing the yield of secondary metabolites and juice production, as well as, for pasteurizing liquid foods without applying heat (Heinz et al., 2001; Saldana et al., 2010; Knorr et al., 2011; Jaeger et al., 2012). Energy inputs of 10–100 J kg^−1^ and pulse widths in the range of 1–100 μs are applied, with time between pulses being considerably longer than the actual pulse time (Knorr et al., 2011; Jaeger et al., 2014b). Although PEF treatment can principally be a non-thermal process, an energy dependent increase in temperature of the treated medium will occur (Van Loey et al., 2001). This phenomenon is termed joule effect (Spilimbergo et al., 2003). A great advantage of the PEF technology is that thermal impact on foods can be reduced. This may lead to fresh-like products with more natural color, texture, taste, and a higher concentration of secondary plant products while the same shelf life can be obtained. However, only a limited inactivation of enzymes is achievable with this treatment, although this problem can be solved by a combination of PEF and other enzyme-inactivating processes (Van Loey et al., 2001; Buckow et al., 2012; Meneses et al., 2013).

PEF treatment is only suitable for liquid foods and its effectiveness is strongly influenced by the electrical conductivity of the product (Devlieghere et al., 2004). The extent of the warming during the treatment depends on the energy input (Van Loey et al., 2001). To achieve consistent results it is important that the distribution of the electric field and consequently the distribution of the temperature field in the treatment chamber are as homogenous as possible (Meneses et al., 2011a; Knoerzer et al., 2012). Numerical simulations of a co-linear chamber done by Meneses et al. (2011a) revealed that temperatures directly on the inside of the electrodes were significantly higher than in-between. As a matter of fact the temperature peaks were about 30–50 K higher than the average temperature. Furthermore, the temperature at the electrode downstream was significantly higher than those at the upstream electrode. The temperature peaks showed considerable maxima and were only obtained for very short times and afterwards declined exponentially (Meneses et al., 2011a).

The effectiveness of PEF treatment concerning inactivation of microorganisms, depends on certain parameters of the treated liquid. One of the most significant parameters is the conductivity σ [S cm^−1^]. The higher ion concentration a liquid contains, the higher is its conductivity. In terms of PEF treatment, high conductivities are counterproductive, since thereby only smaller field strengths can be built up and consequently critical field intensities for cell permeabilization are more difficult to attain (Jayaram, 2000; Toepfl et al., 2007). Furthermore, conductivity depends on temperature, whereas an increase of temperature evokes an increase of electrical conductivity. As temperature rises during PEF treatment higher conductivities will be implicated.

Up to the present the inactivation of vegetative microorganisms by PEFs is well understood and numerous publications on this topic have been published (Toepfl et al., 2007; Jaeger et al., 2014b). However, only limited data on the impact of PEF treatment on bacterial endospores has been published. Several articles showed that PEF processing does not induce any spore inactivation (Wouters et al., 2001; Devlieghere et al., 2004; Knorr et al., 2011), whereas other publications describe a limited inactivation. Other authors have even showed up to 5 log of spore inactivation Spilimbergo et al. (2003) observed 0.5 log_10_ inactivation of *B. cereus* spores with a field intensity of 25 kV cm^−1^, pulse number of 20 at 40°C. A pulse number and field intensity dependent inactivation of *B. cereus* and *B. subtilis* spores at maximally 25°C was described by Marquez et al. (1997). At field strength of 50 kV cm^−1^ and 30 applied pulses 3.5 log_10_ of *B. subtilis* spores were inactivated. A pulse number of 50 led to a 5 log_10_ inactivation of *B. cereus* spores. Most of the published data were obtained under different treatment conditions and set-ups and thus the comparison between them is not possible. However, a certain level of spore inactivation could be achieved if the necessary harsh conditions are applied.

Therefore, the impact of different PEF parameters on microbial inactivation as well as the synergistic effect of the electric field and heat was investigated in this study.

## Materials and methods

### Spore strains and preparation

The sporulation of the strain *B. subtilis* PS832 was induced in accordance with a method described elsewhere (Nicholson and Setlow, 1990). One single colony of *B. subtilis* PS832, cultivated on nutrient agar plates (Oxoid Ltd., Basingstoke, UK) was transferred to nutrient broth (Oxoid Ltd., Basingstoke, UK) and incubated for 24 h at 37°C. 100 μL cell suspension was plated on 2xSG agar plates and incubated at 37°C for 7 days. Spores were harvested and cleaned by repeated centrifugation (5000 g) after minimum of 95% of the spore population turned on to a bright phase. Finally the suspension was washed with cold distilled water. The cleaned spore suspension contained approximately 2.5 × 10^9^ spores ml^−1^.

*G. stearothermophilus* ATCC 7953 (Merck Sterikon® plus Bioindikator, Merck KGaA, Darmstadt, Germany) was used as a thermal sterilization indicator microorganism with certified heat resistance (D_121° C_ = 1.6 min). The used spore suspension contained approximately 1.0 × 10^6^ spores ml^−1^.

### Thermal endospore inactivation

*B. subtilis* and *G. stearothermophilus* spore suspensions were diluted 1:10 in saline solution with an electrical conductivity of 5.3 mS cm^−1^, skim milk (5.3 mS cm^−1^, 0.3% fat) and fresh prepared and filtered carrot juice (7.73 mS cm^−1^). These suspensions were filled in thin glass capillaries with an internal diameter of 1 mm, an external diameter of 1.3 mm and a length of 100 mm (Kleinfeld Labortechnik GmbH, Gehrden, Germany) and heat sealed. Thermal inactivation was done in a thermostatic bath (Polystat K6, Huber GmbH, Offenburg, Germany) at 95, 100, 105, and 110°C for *B. subtilis* and 115, 121, and 130°C for *G. stearothermophilus* at different treatment times. The heat-up time to reach isothermal temperatures in the center of the capillary at 130°C was 7.5 s (Mathys, 2008). Glass capillaries were rapidly cooled in ice water immediately after the heat treatment was performed. The achieved inactivation was determined by plating appropriate dilutions on nutrient agar plates (CM0003, Oxoid Ltd., Basingstoke, UK). After incubation at 37°C survivors were enumerated after 24 and 48 h. The PEF treated spore suspensions were collected, diluted, plated on Nutrient Agar plates and incubated as described above.

All treatments were done in duplicates.

### Setup of the used PEF equipment

**Figure 1:**
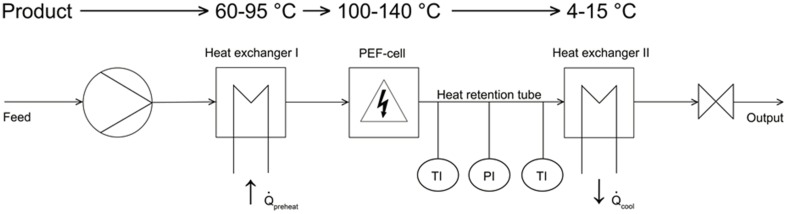
**Schematic drawing of the used PEF equipment**.

The PEF experiments were conducted by the use of continuous PEF equipment shown in Figure 1, whereas the first component was a laboratory-scale progressive cavity pump (Hanning Elektro-Werke GmbH & Co. KG, Oerlinghausen, Germany) operating at flow rates of 5–7 l h^−1^.

A stainless steel tempering coil (Technische Universitaet Berlin) was used to preheat the different liquids at the desired inlet temperatures (70°C and 80°C for *B. subtilis* and 95°C for G. *stearothermophilus*). Downstream of the preheating zone, the spore suspension entered into a co-linear treatment chamber, which is described in detail by Meneses et al. (2011a).

**Figure 2:**
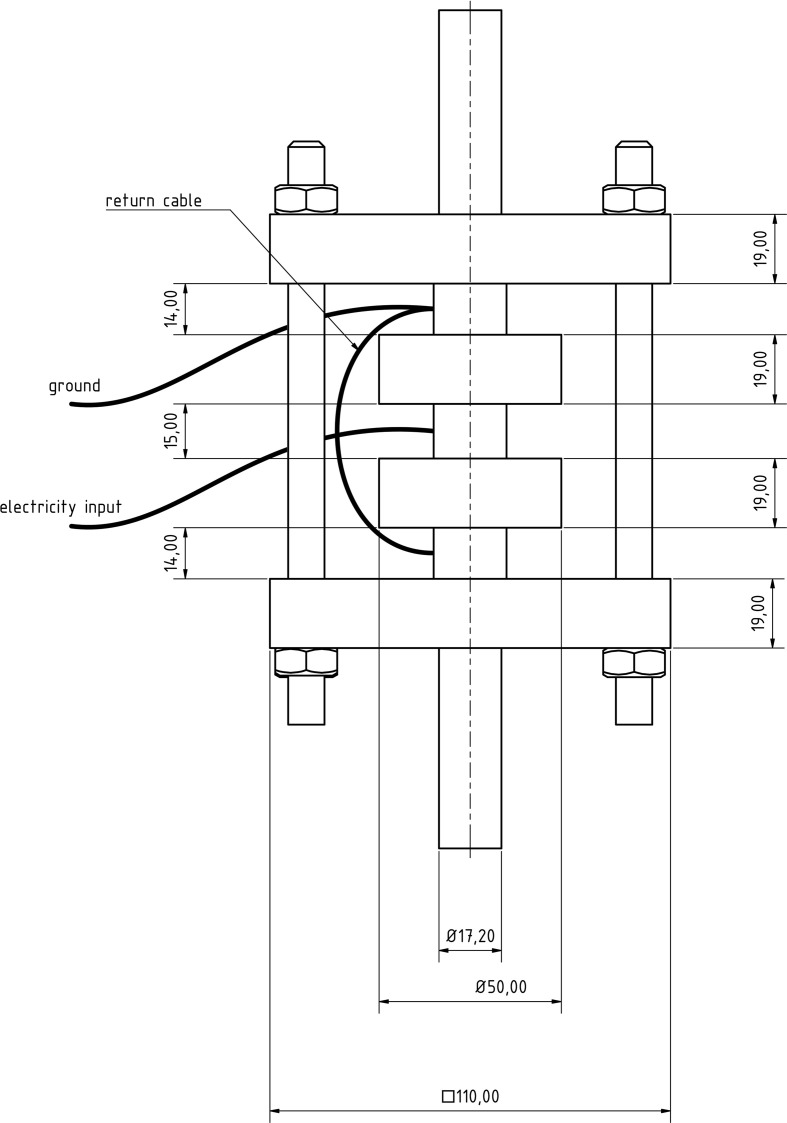
**Dimensioning (in mm) and wiring of the used co-linear PEF treatment chamber**.

The PEF cell (Figure 2) consisted of two quadratic Teflon® top- and bottom insulators, one at each end of the cell, followed by one cylindrical stainless steel grounded electrode, respectively. A return cable connected both grounded electrodes. The center of the treatment chamber was formed by a single cylindrical stainless steel high voltage electrode, surrounded by two cylindrical Teflon® insulators, one at each side of this electrode. The inner shape of the used insulators was convex as described by Meneses et al. (2011c).

For the continuous PEF treatment a 7 kW pulse modulator (ScandiNova Systems AB, Uppsala, Sweden) providing rectangular pulses in the range of 3–8 μs with a maximum voltage of 50 kV and a maximum repetition rate of 400 Hz was used.

Downstream of the PEF-treatment chamber, the liquid flowed into a 65.5 cm long, isolated heat-retention pipe (inner diameter 1 cm), equipped with a pressure gage (located 37.5 cm downstream) and two K-type thermocouples (located at18 and 57.5 cm downstream). Both thermocouples' tips were placed at the center of the heat-retention pipe. An adjustable throttle at the end of the heat retention unit was used to adjust the pressure up to 5 bar in order to avoid evaporation of the liquid media. A second heat exchanger (identical to the first one) after the throttle served to cool the treated liquids to ambient temperature.

### PEF treatment parameter used for spore inactivation

For the PEF inactivation trials, *B. subtilis* spores were suspended 1:10 in skim milk and saline water. Voltages of 6, 8, and 10 kV (10.26, 13.68, and 17.1 kV/cm) were applied at frequencies (f) between 75 and 300 Hz and pulse width of 8 μs. Specific pulse energies (W_pulse_) of 1–3 J pulse^−1^ were applied, as ascertained by an oscilloscope (TDS 430 A, Tektronix Inc., Beaverton, OR, USA). Volume flows ($\dot{V}$) of 5.1–6.8 l h^−1^ and preheating temperatures (T_in_) of 70 and 80°C were used.

Equation (1) was used to calculate the energy input (W_input_).

(1) $$W_{input}=\frac{f\;\cdot \;W_{pulse}\;\cdot \;3.6}{\dot{V}}$$

Equation (2) was used to estimate the thermal load, with c_p_ as the specific heat capacity of the treatment media and T_in_ as the preheating temperature.

(2) $$Thermal\;load=\frac{W_{inpit}}{c_{p}}+T_{in}$$

The applied PEF treatment parameters resulted in energy inputs of 60.92–257.14 kJ kg^−1^ and thermal loads of 94.61–136.25°C.

For the inactivation of *G. stearothermophilus* spores, the same voltages were used as for *B. subtilis*. Furthermore, the following parameters were applied: frequencies of 50–215 Hz, pulse widths of 4, 6, and 8 μs, volume flows of 3.1–7.3 l h^−1^ and a preheating temperature of 95°C. The following parameters were determined or calculated, respectively: specific pulse energies of 1.2–3.2 J pulse^−1^, energy inputs of 83.08–294.55 kJ kg^−1^ and thermal loads of 115.77–168.64°C. PEF inactivation of these spores was conducted in saline water, skim milk and carrot juice. All treatments were done at least in duplicates.

### Calculation of spore inactivation and numerical simulation

In order to enable a comparison between thermal effects and the combined PEF-thermal effect, *D*- and *z*-values were calculated for the pure thermal treatments. The *D*-value (D_T_, Equation 3) indicates the required time needed for a 90% inactivation at a constant treatment temperature T, where t is the treatment time, N_0_ is the initial spore count and N_t_ is the spore count at time t.

(3) $$D_{T}=\frac{t}{log_{10}N_{0}-log_{10}N_{t}}$$

The *z*-value denotes the temperature increase / decrease, which is necessary to reduce / raise the *D*-value by a factor of ten. It can be calculated with Equation (4), where T is the temperature and, T_0_ is the reference, D_T_ is the *D*-value for the temperature T and D_T0_ is the *D*-value for the reference temperature T_0_.

(4) $$z=\frac{T-T_{0}}{log_{10}D_{T_{0}}-log_{10}D_{t}}$$

The *F*-value also considers non-constant temperatures that have an influence on the bacterial count and is therefore used as a reference for the effectivity of sterilization processes, including preheating and cooling. A reference temperature T_0_ of 121.1°C was used. The *F*-value was calculated with Equation (5), where T(t) is the temperature profile, z is the *z*-value, t_1_ and t_2_ are the start and final temperatures, respectively.

(5) $$F_{T_{0}}=\int_{t_{1}}^{t_{2}}10^{\left\lceil \frac{T(t)-T_{0}}{z}\right\rceil }$$

The calculation of the inactivation levels for the PEF treated spores was considerably more complex than the above described linear inactivation due to extremely inhomogeneous temperature distribution in the treatment chamber (Meneses et al., 2011c). To calculate these inhomogeneous temperature fields numerical computational fluid dynamics (CFD) simulations were necessary as described elsewhere (Fiala et al., 2001; Lindgren et al., 2002; van den Bosch et al., 2003; Gerlach et al., 2008; Jaeger et al., 2009; Buckow et al., 2010; Meneses et al., 2011c). Based on the calculated temperature profiles for the wall and center of the treatment chamber a solely thermal inactivation (*F*-value) was calculated for each kinetic data point, based on the *D*- and *z*-values, which were determined during thermal inactivation in glass capillaries. The *F*-values for the wall- and center temperatures were arithmetically averaged and the obtained F_average_ was used for further comparison. Finally, the solely thermal inactivation that was achieved during the PEF treatment was calculated with Equation (6) and compared to the experimentally determined total inactivation of the PEF process. This enabled to distinguish possible synergistic effects between PEF-treatment and temperature effects.

(6) $$\text{log}\left(\frac{\text{N}}{{\text{N}}_{0}}\right)=\frac{{\text{F}}_{\text{average}}}{{\text{D}}_{\text{reference}}}$$

All inactivation data were normalized and logarithmized to log(N· N^−1^_0_) and finally plotted with Origin® 8 to obtain a log(N· N^−1^_0_) over frequency diagram.

## Results

### Characterization of the system and used PEF parameters

**Figure 3:**
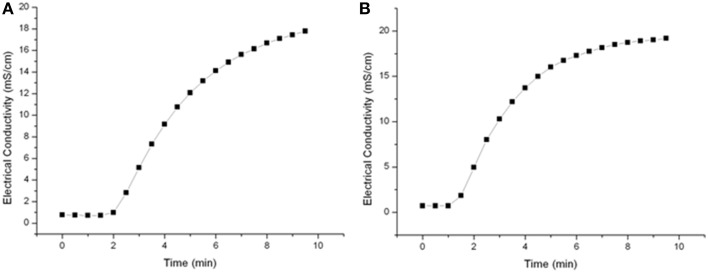
**Residence time spectra for a 4.8 l h^−1^ (A) and 7 l h^−1^ (B) flow rate**.

A residence time spectrum was measured in order to determine the right timing for sampling or switching to a new PEF-parameters set up. Saline water (20 mS cm^−1^) was circulated within the PEF-system at throughputs of 4.8 l h^−1^ or 7 l h^−1^ and samples were taken every 30 s. The calculated flow rate for these throughputs is in between 1.7 and 2.48 cm s^−1^ resulting in mean holding times in the heat-retention pipe of 26–38.5 s. According to Figure 3, the higher flow rate did not significantly influence sampling time and hence, 7 min was selected as sampling time after parameter or medium was changed.

Further it was detected how long it took to gather stable system temperatures in the heat retention tube after changing the preheating temperature or the PEF-treatment parameter. After constant treatment conditions were achieved, the system equilibrated within 3 min.

### Thermal inactivation

The inactivation in glass capillaries related to only thermal effects and the respective *D*- and *z*-values are shown in Table 1. The calculation of each *D*-value is based on at least 5 kinetic points. For the inactivation of *B. subtilis*, *z*-values of 7.41°C (saline water) to 8.04°C (skim milk) were obtained. For the thermal inactivation of *G. stearothermophilus z*-values of 17.83 and 18.25°C for saline water and skim milk, as well as 10.52°C for carrot juice were obtained.

**Table 1:** **Thermal inactivation data for *B. subtilis* and *G. stearothermophilus* spores in various treatment media and their corresponding *D*-, and *z*-values**.

**Strain**	**Medium**	**Temperature [°C]**	***D*-value [min]**	***z*-value [°C]**
*B. subtilis*	Saline water	95	23.64	7.41
		100	3.69	
		105	0.93	
		110	0.21	
	Carrot juice	95	11.70	7.96
		100	1.76	
		105	0.53	
		110	0.14	
	Skim milk	95	13.44	8.04
		100	2.47	
		105	0.60	
		110	0.18	
*G. stearothermophilus*	Saline water	115	5.53	17.83
		121	1.88	
		130	0.77	
	Carrot juice	115	6.93	10.52
		121	1.46	
		130	0.25	

### Impact of different PEF parameters on spore inactivation

**Figure 4:**
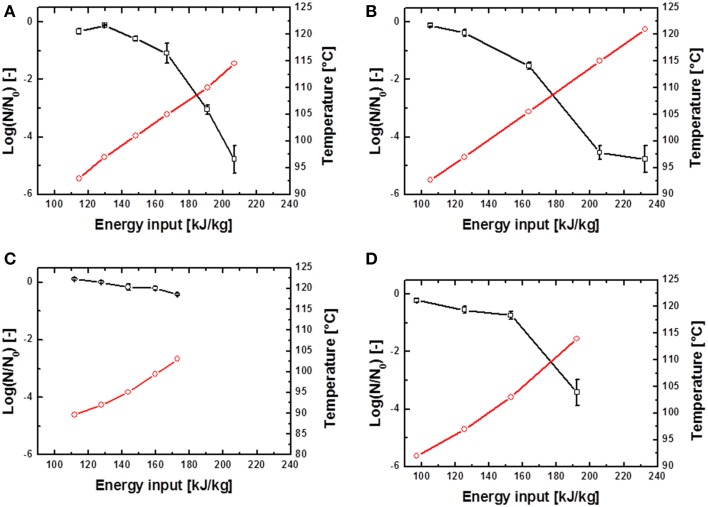
**Inactivation data for *B. subtilis* in saline water (A,B) and skimmed milk (C,D)**. Preheated to 70°C and treated with *U*_0_ = 6 kV; 10.26 kV/cm **(A,C)** and *U*_0_ = 8 kV; 13.68 kV/cm **(A,C)**. 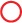 represents the temperature T_out,a_ 18 cm above the PEF-treatment zone and 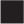 the achieved spore inactivation log(N/N_0_).

**Figure 5:**
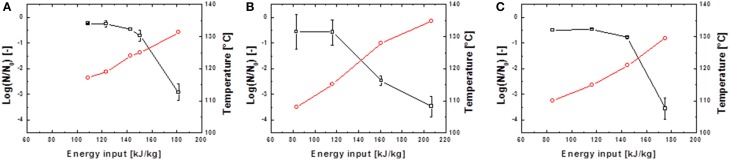
**Inactivation data for *G. stearothermophilus* in saline water (A) and skimmed milk (B) and carrot juice (C)**. Preheated to 95°C and treated with *U*_0_ = 8 kV; 13.68 kV/cm. 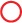 represents the temperature T_out,a_ 18 cm above the PEF-treatment zone and 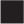 the achieved spore inactivation log(N/N_0_).

The inactivation of spores was conducted at different energy inputs, starting with relatively low values of averagely 80 kJ kg^−1^. W_input_ was increased successively until electrical arcs occurred. Maximum energy inputs of averagely 250 kJ kg^−1^ were obtained. Exemplary PEF inactivation curves of *B. subtilis* and *G. stearothermophilus* are displayed in Figures 4, 5 and discussed in further detail hereafter. Considering the different inactivation curves it has to be mentioned that the extent of both, W_input_ as well as T_out,a_ (measured 18 cm downstream of the PEF treatment zone) is directly proportional, so that in the following graphs only T_out,a_ is displayed.

In case of the spore inactivation by PEFs it is obvious that an increase of the energy input leads to higher temperatures and therefore to an increase in spore inactivation. Some of the inactivation kinetics displayed a distinct shoulder at the beginning of the treatment. After the shoulder, a mostly linear progression of the inactivation curve occurred. Furthermore, some inactivation graphs exhibited a tailing at high energy inputs.

By comparison of different inactivation curves it is notable that data points of similar PEF parameter settings and equal frequencies displayed higher inactivation rates for higher preheating temperatures due to slightly higher energy inputs (Table 2). Furthermore, an increase of each energy-input influencing parameter (Equation 1) leads to an increase of W_input_ and therefore to higher inactivation rates. Exemplarily figures Figures 4 A,B signify that higher charging voltages lead to an increased spore inactivation for identical frequencies and comparable parameters settings. In order to evaluate the effects of the respective PEF parameters on spore inactivation representative data points were selected that exhibited similar energy inputs and consequently similar temperatures (Table 2). To gather reliable and statistically safe results, data points with inactivation levels of at least 1.5 log_10_ were predominantly considered. In the range of these points linear inactivation kinetics were assumed.

**Table 2:** **Parameter sets for the analysis of PEF parameter dependent spore inactivation**.

**Species**	**Matrix**	***U*_0_ [kV]**	***f* [Hz]**	***t* [μs]**	***W*_pulse_ [J/pluse]**	**$\dot{V}$[l/h]**	***T*_preheat_ [°C]**	***T*_out, a_ [°C]**	***W*_input_ [kJ/kg]**	**log [N/N_0_]**
*B. subtilis*	Saline water	6	225	8	1.4	6.8	70	105.0	166.7	1.1 ± 0.3
		8	125		2.4	6.6		105.5	163.6	1.5 ± 0.1
		6	250		1.2	6.4	80	112.4	163.1	3.5 ± 0.3
		8	150		2.0	6.4		113.8	168.8	3.4 ± 0.5
		10	100		3.0	6.0		115.9	180.0	3.5 ± 0.3
	Skim milk	6	250		1.0	6.3	70	99.4	142.9	0.2 ± 0.5
		10	75		2.9	5.9		99.4	132.7	0.2 ± 0.2
		6	250		1.1	6.2	80	110.5	159.7	3.0 ± 0.4
		10	100		2.5	5.6		111.0	160.7	3.0 ± 0.7
*G. stearother- mophilus*	Saline water	6	200	6	1.5	6	95	131.7	180.0	3.2 ± 0.4
		8	175	4	1.7	5.9		131.3	181.5	2.9 ± 0.3
		10	100		2.7	5.5		132.4	176.7	2.2 ± 0.5
	Skim milk	6	125	6	1.5	3.1		138.0	217.7	2.7 ± 0.6
		8	200		1.6	5.5		130.6	209.5	2.5 ± 0.7
		10	75	4	3.1	4.6		132.7	182.0	3.1 ± 0.4
	Carrot juice	6	215		1.3	4.9		135.0	205.4	3.4 ± 0.4
		8	125		2.1	5.4		129.5	175.0	3.5 ± 0.5

It is evident that in almost all cases similar energy inputs led to comparable inactivation levels. Only for the inactivation of *B. subtilis* spores in saline water and high electric field strength slight deviations occurred. For the inactivation of *G. stearothermophilus* in saline water the lowest inactivation of 2.2 log_10_ was achieved at the lowest energy input of 176.7 kJ kg^−1^ and the lowest frequency of 100 Hz although this sample was treated with the highest temperature (T_out,a_) of 132.4°C. On the other hand, *G. stearothermophilus* spores suspended in skimmed milk exhibited the highest inactivation of 3.1 log_10_ with an intermediate temperature of 132.7°C, the lowest frequency of 75 Hz and the lowest energy input of 182 kJ kg^−1^. Since these anomalies only occurred for two data points it is most likely that the altered inactivation is caused by other factors, like electrical arcing.

The inactivation of spores in carrot juice was more difficult since the higher electrical conductivity led to earlier electrical arching and therefore only lower energy inputs could be achieved. Due to the higher thermal resistance of *G. stearothermophilus* (Table 1), higher temperatures and therefore higher energy inputs were necessary to achieve an inactivation comparable to that of *B. subtilis*.

### Beneficial effects of PEF treatment on spore inactivation

To estimate whether a combined PEF-thermal treatment has a beneficial or possible a synergistic effect on spore inactivation compared to a pure thermal inactivation, CFD modeling coupled with the determined thermal inactivation data was used.

**Figure 6:**
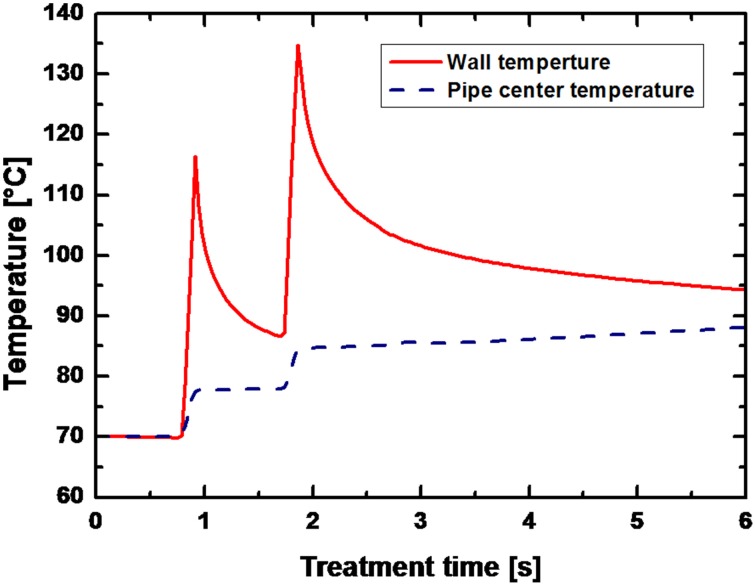
**Temperature field in the PEF chamber for *B. subtilis* spores suspended in saline water**. Process parameters: *U*_0_ = 8 kV (13.68 kV/cm), *f* = 150 Hz, τ = 8 μs, *W*_pulse_ = 2.6 J pulse^−1^, *V* = 6.2 l h^−1^, *T*_preheat_ = 70°C, *W*_input_ = 226.5 kJ kg^−1^. The solid line represents the wall temperature and the dashed line the temperature in the center of the pipe.

For low energy inputs, temperatures of 90°C were reached after averagely 1.5 s at a preheating temperature of 70°C and temperatures of 110°C were reached after approximately 2 s at a preheating temperature of 95°C (data not shown). An exemplary temperature profile within the PEF-treatment chamber (only the temperatures at the wall and in the center are given) is shown in Figure 6.

Within the first PEF-treatment zone (after 0.91 s) a peak temperature of 116.3°C was calculated at the pipe wall, whereas the temperature in the center of the pipe remained at 77.5 C. In the second PEF-treatment zone this temperature difference was even more pronounced with 134.7°C at the wall and 84.6°C after 1.85 s.

The results of the temperature field simulated within the PEF treatment chamber were used as inputs of Equation (5) (*F*-value) and thus, the spore inactivation related to pure thermal effects were calculated. To consider the possible deviations of the simulated temperature with the measured temperatures we assumed the simulation may fail from reality within ±2°C, according to Jaeger et al. (2009). The calculated *F*-values gave theoretical range of thermal only inactivation in an identical temperature field without PEF application (Table 3) and enabled a comparison of theoretical thermal inactivation results with the achieved experimental inactivation by a combined PEF-thermal treatment.

**Table 3:** **Comparison of combined PEF-thermal inactivation (experimental data) and thermal only spore inactivation (calculated values)**.

**Strain**	**Medium**	***U* [kV]**	***f* [Hz]**	***W*_input_ [kJ/kg]**	***T*_out,a_ [°C]**	**PEF-thermal inactivation (measured)**	**Thermal-only inactivation (theoretical)**	**Δ log**
*B. subtilis*	Saline water	8	140	188.1	109	3.2	1.6-5.5	Not possible to determine
			150	226.5	117	4.7	2.0-6.9	Not possible to determine
	Skim milk	13	90	130.0	121	5.0	1.9–6.5	Not possible to determine
*G. stearothemo-philus*	Saline water	6	125	118.4	118	3.3	0.0-0.1	3.2–3.3
		10	60	144	122	3.0	0.1–0.4	2.6–2.9
	Skim milk	13	90	147	126	3.7	0.4–1.3	2.4–3.3

*The given theoretical values for a thermal only inactivation represents ranges of inactivation assuming a simulated temperature ±2°C for an identical temperature field without PEF application*.

The difference between both treatments (Δ log) shows the additional spore inactivation achieved by combining PEF and thermal effects. In case of *B. subtilis* no difference to a pure thermal inactivation could be verified, which might be due to the high energy input in the combined PEF-thermal treatment. However, for *G. stearothermophilus*, an additional inactivation of 2.4–3.2 log_10_ presumably caused be the PEF was calculated. The accelerated inactivation of *G. stearothermophilus* spores is consequently considerably higher than the inactivation of *B. subtilis* spores.

## Discussion

To characterize the used PEF system and to ensure reproducible sampling, residence time spectra for different flow rates were determined. The tested flow rates of 4.8 and 7.1 l h^−1^ had nearly no impact on the residence time distribution (Figure 3) and hence 7 min was selected as sampling time after parameter or medium change was done.

During the trials, energy inputs were successively increased for each experimental trial until electrical breakdowns occurred. This phenomenon emerged earlier for higher preheating temperatures and higher electrical conductivities. The used carrot juice exhibited an electrical conductivity of 7.73 mS cm^−1^ and therefore PEF processing was more difficult due to earlier appearing electrical breakdowns. For this reason this medium was processed with pulse widths of only 4 μs (Table 2). It was also noticeable that in carrot juice the actually rectangular pulse shape changed to an exponentially declining waveform.

To estimate if a combined PEF-thermal treatment has a beneficial effect on spore inactivation liquid media, *D*- and *z*-values for each spore strain in each media were determined (Table 1). With the used method, we could confirm a D_121° C_-value of 1.6 min given in certificate for *G. stearothermophilus*. The reason for variations of the *D*- and *z*-values for *B. subtilis* and *G. stearothermophilus* in the different treatment media, could be explained due to matrix effects. The small solid sediment particles that were present in the carrot juice most probably exerted a protective effect on the spores. The reason for this behavior is the lower heat conductivity of the solids in comparison to the aqueous continuous phase. The deteriorated inactivation of the *B. subtilis* spores in milk can be explained by a protective effect of the milk fat (Kessler, 2002). However, the obtained *D*- and *z*-values correspond to literature data (Reineke et al., 2011). Dogan et al. (2009) reported a D_121° C_ of 0.7 min and a *z*-value of 13°C in Ringer's solution, whereas Somavat et al. (2012) determined a D_121° C_ of 2.53 min, a D_130° C_ of 0.6 min as well as a *z*-value of 7.42°C in tomato soup.

In contrast to the nearly linear progress of spore inactivation kinetics in thin glass capillaries, the kinetics for a combined PEF-thermal treatment were non-linear. The shoulder formation in Figures 4, 5 can be explained by the disaggregation of spore agglomerates (Mathys et al., 2007). The occurring tailing in some kinetics can be explained by different resistances of spores among a certain population and the accumulation of more resistant spores toward the end of the treatment. However, in case of the executed experiments the more plausible explanation is that the electrical breakdowns at high energy inputs, which occurred at the end of almost every treatment, led to an altered flow of the electric current through the electrode-surrounding air rather than through the treatment medium and therefore lower inactivation rates were achieved.

Further it can be concluded, that within the tested range of PEF-parameters no parameter solely executed a significant influence on the inactivation of spores (Table 2, Figures 4, 5). However, other, non-electrical parameters like preheating temperature or the respective volume flow had an influence on spore inactivation. Moreover, the pH-value can alter significantly during PEF treatment (Meneses et al., 2011b) and therefore an additional inactivation of spores can occur. Similar findings were reported by Siemer et al. (2014a). They reported a 1.6 log_10_ inactivation of *B. subtilis* spores using 167 kJ kg^−1^ in Ringer's solution with pH 4 compared to a 0.6 log_10_ reduction in Ringer's solution with neutral pH. Further, under identical process conditions (80°C preheating and 9 kV cm^−1^ electric field strength) the addition of 10% sugar permitted to reduce the needed energy input to achieve a 3 log_10_ reduction from 178 to 146 kJ kg^−1^.

Somavat et al. (2012) showed a dependence of spore inactivation on electric parameters for ohmic heating. A higher inactivation was found for *G. stearothermophilus* spores treated at 10 kHz compared to samples treated at 60 Hz and therefore revealed a significant influence of the frequency. It is possible that these results can also be applied for PEF treatment, although this hypothesis was not proven by the executed experiments and therefore has to be verified with specific tests.

However, the dominant impact on spore inactivation was the preheating and maximum treatment temperature (Table 2, Figures 4, 5). Whereas, Bermúdez-Aguirre et al. (2012) reported a higher resistance of PEF treated *B. cereus* spores in milk at ambient temperatures compared to a preheating to 40°C. Temperatures of 50°C resulted in an increased inactivation of 3 log_10_. This treatment was accomplished at field intensities of 35 kV cm^−1^ and 10 pulses. Besides, a utilization of skim milk led to higher inactivation rates compared to a treatment in whole milk (Bermúdez-Aguirre et al., 2012). An explanation for this behavior could be a possible spore-protective effect of the milk fat. Further, Somavat et al. (2012) described synergistic effects of electricity and heat to *G. stearothermophilus* spores by use of ohmic heating at 10 and 60 Hz. At this a maximum inactivation was achieved by an application of 130°C and 60 Hz.

Considering the contrastive statements mentioned above it can generally be stated that spores are significantly more resistant to PEF treatment than vegetative cells. A possible reason for this phenomenon could be the considerably lower electrical conductivity of spores due to the low water content and immobilized ions in the core, as well as their much smaller cross-section, which makes the spores less susceptible to the electric field (Wouters et al., 2001; Jaeger et al., 2014b). However, since application of PEFs is always associated with a temperature dependent heating of the treatment medium, the temperature field in the PEF chamber has always to be taken into account (Meneses, 2011).

Since the aim of this study was to inactivate spores, relatively high energy inputs were necessary and consequently high temperatures emerged (Figure 6). Due to the utilization of a co-linear treatment chamber, inhomogeneous temperature fields occurred with considerable temperature differences between the wall and the center of the treatment chamber (Meneses et al., 2011a). In UHT processes for the preservation of milk temperatures of approximately 140°C are used and applied for circa 4 s (Kessler, 2002). For a solely thermal sterilization of liquid foods by PEFs similar temperature-time regimes have to be achieved. In case of the examined data points temperature peaks of maximum 60 K above the preheating temperature could be achieved between wall and center of the treatment chamber, leading to temperature increases of the treatment liquid of averagely 20 K (Figure 6).

To verify if a combined PEF-thermal treatment has a beneficial effect on spore inactivation, numerical simulations were used, which coupled the reference data for a pure thermal inactivation with the spatial temperature field in the PEF-chamber. The results presented in Table 3 shows no impact on *B. subtilis* inactivation but an additional or possibly synergistic effect of the PEF treatment on the inactivation of *G. stearothermophilus* spores.

To consider the possible deviations of the simulated temperature with the measured temperatures we assumed the simulation may fail from reality within ±2°C. Thus, the inactivation was in some cases higher than the experimental one and the calculated theoretical inactivation varies enormous at high energy inputs. Hence, in some cases it was not possible to distinguish between PEF and thermal inactivation as the theoretical inactivation was higher than the experimental one.

For *B. subtilis* spore inactivation the specific energy input was significantly higher than for *G. stearothermophilus*, but much lower inactivation rates were achieved (Table 3), even if the additional 105 kJ kg^−1^ needed to reach 95°C pre-heating temperature are taking into account.

Consequently it is likely that the higher temperatures for the inactivation of *G. stearothermophilus* contributed to its susceptibility toward the electric field. It could also be possible that the structure of *G. stearothermophilus* spores is responsible for its greater electrical vulnerability but this is unlikely due to significant similarities of both used spore types (Setlow, 2007). The obtained results imply that the inactivation of bacterial endospores by a combined PEF-thermal process is not solely based on thermal effects, so that it can be concluded that the electric field generates an additional inactivation. This was also reported by Siemer et al. (2014b), who reported an increased total spore inactivation for higher energy inputs (up to 195 kJ kg^−1^) for a combined PEF-thermal treatment. After applying 195 kJ kg^−1^ to *B. subtilis* spores suspended in Ringer's solution (pH 7) a 4.4 log_10_ inactivation was achieved, which could be separated in 1.15 log_10_ of thermal and 3.25 log_10_ of PEF related inactivation. These data are, with regard to the high energy input and the achieved inactivation, in agreement with our data (Table 3).

However, the exact mechanism of the accelerated spore inactivation due to the electric field is completely unclear. Somavat et al. (2012) hypothesized that the accelerated inactivation is achieved by the rotation of temperature-released polar spore components in the electric field. However, it is most likely that this explanation is inappropriate, since it is generally accepted that temperatures that trigger the release of spore components are so high that at this point essential spore components are already denatured so that the spore is already lethally damaged (Coleman et al., 2007). A possible explanation for this synergism could be the greater resistive heating of the spore's core due to its very low electrical conductivity (Bassi et al., 2012) and higher electric resistance. Consequently the core could undergo increased heating in comparison to the surrounding medium. Nevertheless, this theory cannot explain the high inactivation at higher temperatures that was described above. Another reason for this phenomenon could be a possible detachment of ions from the inner spore membrane and the subsequent migration of these ions through the spore's core. This could lead to a possible reduction of the inner spore membrane's barrier function and therefore to a greater susceptibility of the spore. However, the above-mentioned explanations are only hypotheses. The investigation of the exact spore inactivation mechanism by PEFs needs further detailed research.

## Conclusion

In conclusion, it can be stated that a combined application of PEFs and thermal treatment can lead to an accelerated inactivation of endospores in comparison to a pure thermal inactivation in an identical temperature field. It is most likely that the additional sporicidal effect of the electric field is temperature dependent, although the reason for this phenomenon is not clarified yet. To investigate this, quantification of possibly leaked dipicolinic acid from the spore core, flow cytometry and high resolution microscopy might be suitable tools. Additionally it is necessary to analyze the influence of the required high energy inputs on other food constituents in order to determine whether desired or undesired modifications have emerged, to use this treatment as an alternative technique of ultra-high temperature processing for liquid foods with a high pH-value like milk, vegetable juices or soups.

### Conflict of interest statement

The authors declare that the research was conducted in the absence of any commercial or financial relationships that could be construed as a potential conflict of interest.
